# The experimental study on susceptibility of common European songbirds to *Plasmodium elongatum* (lineage pGRW6), a widespread avian malaria parasite

**DOI:** 10.1186/s12936-019-2926-4

**Published:** 2019-08-27

**Authors:** Mikas Ilgūnas, Vaidas Palinauskas, Elena Platonova, Tatjana Iezhova, Gediminas Valkiūnas

**Affiliations:** Nature Research Centre, Akademijos 2, 08412 Vilnius, Lithuania

**Keywords:** Avian malaria, *Plasmodium*, *Plasmodium elongatum*, Birds, Phanerozoites, Pathology

## Abstract

**Background:**

*Plasmodium elongatum* (cytochrome *b* lineage pGRW6) is a widespread avian malaria parasite, often causing severe disease in non-adapted hosts. This parasite lineage is of global distribution however, its virulence remains insufficiently understood, particularly in wild birds. Surprisingly, this infection has never been reported in Common starlings *Sturnus vulgaris* and Common crossbills *Loxia curvirostra*, common European songbirds which were extensively sampled across Europe. A hypothesis was proposed that these birds might be resistant to the pGRW6 infection. The aim of this study was to test this hypothesis.

**Methods:**

Lineage pGRW6 was isolated from a naturally infected Eurasian reed warbler, multiplied in vivo and inoculated in Common starlings and Common crossbills. Experimental and control groups (8 birds in each) were maintained in controlled conditions and examined microscopically every 4 days. Haematocrit value and body mass were monitored in parallel. At the end of the experiment (44 days post exposure), samples of internal organs were collected and examined using histological methods for possible presence of phanerozoites.

**Results:**

All control birds remained uninfected. Experimental starlings were resistant. All exposed crossbills were susceptible and survived until the end of this study. Prepatent period was 12–16 days post exposure. Light parasitaemia (< 0.7%) developed in all birds, and only few phanerozoites were seen in bone marrow cells of 5 of 8 experimentally infected crossbills. Significant changes were reported only in haematocrit value but not body mass in the exposed crossbills compared to controls.

**Conclusion:**

*Plasmodium elongatum* (pGRW6) is of low virulence in Common crossbills and is unable to develop in Common starlings, indicating innate resistance of the later bird species. Low virulence in Common crossbills is likely due to the inability or low ability of this parasite lineage to develop phanerozoites resulting in light (if at all) damage of stem bone marrow cells. This study suggests that susceptibility of different bird species to the lineage pGRW6 is markedly variable. The global distribution of this parasite might be due to low virulence in wild adapted avian hosts, which survive this infection and serve as reservoirs host for non-adapted birds in whom this infection is often lethal.

## Background

Malaria is burdening birds worldwide. In all, 55 morphologically readily distinct species of avian *Plasmodium* have been identified [1] and genetic data suggest that their number might be even greater [2]. However, virulence of the majority of *Plasmodium* infections remain insufficiently investigated, particularly in wildlife. Severe disease and mortality due to malaria have been often reported in zoos, aviaries and private collections worldwide. Non-adapted wild bird species also suffer dramatically [3–7]. Information about malaria influence on adapted wild birds is contradictory, with no certain pattern during development of the same *Plasmodium* species lineage in different species of avian hosts. For example, experimental data indicate that the cytochrome *b* lineage (*cytb*) pSGS1 of *Plasmodium relictum* might develop high parasitaemia resulting in severe anaemia in Common crossbills *Loxia curvirostra*, Eurasian siskins *Carduelis spinus* [8], but not in House sparrows *Passer domesticus* and Common chaffinches *Fringilla coelebs* [9]. The same is true for exo-erythrocytic development of the parasites. Mainly, *cytb* lineage pCOLL4 of *P. homocircumflexum* demonstrates markedly different ability to produce phanerozoites in different species of avian hosts resulting in different virulence and mortality rates [10].

*Plasmodium elongatum* (*cytb* lineage pGRW6) is one of the most widespread avian malaria agents, which has been reported in birds belonging to more than 15 avian families and 11 orders [2]. This parasite species was discovered by Clay G. Huff in 1930 in the USA, where it is widespread and prevalent. Since then, this infection has been reported in all continents, except Antarctica [11, 12]. However, intraspecies genetic variation of this pathogen was unclear. The lineage pGRW6 was originally determined by Beadell et al. [13], and it was assigned to *P. elongatum* approximately 10 years ago [14]. Several closely related lineages are available in GenBank and MalAvi database, and they probably belong to *P. elongatum*, but morphological evidence is lacking except for the lineage pERIRUB01. For unclear reasons, the latter parasite lineage is rare in wildlife [15].

*Plasmodium elongatum* was known to cause severe disease and even death in captive zoo birds around the world already during the “pre-molecular era” [16–21]. Parasitaemia is usually light (< 1%) both in naturally and experimentally infected birds [11, 16]. Thus, the primary reason of disease and death has been linked to distortion of stem bone marrow cells by exoerythrocytic meronts (phanerozoites) [11, 16]. Phanerozoites develop primarily in cells of the haemopoietic system, particularly in bone marrow, often resulting in the disruption of erythropoiesis and leading to anaemia even during light parasitaemia [12, 16, 22]. Recent molecular studies show that *P. elongatum* (pGRW6) is responsible for severe disease and even mortality in captive and wild non-adapted bird species worldwide [4–6].

Surprisingly, *P. elongatum* (pGRW6) infection has never been reported in Common starlings *Sturnus vulgaris* or Common crossbills *Loxia curvirostra*, common Holarctic songbirds which have been extensively sampled for haemosporidian parasites across Europe [2]. There are records of malaria parasites and other related haemosporidians in these two bird species (*Plasmodium ashfordi* lineage pGRW2), *Plasmodium relictum* (lineages pSGS1 and pGRW4), *Plasmodium homocircumflexum* (lineage pCOLL4), *Plasmodium unalis* (lineage pTUMIG03), *Haemoproteus tartakovskyi* (lineage hSISKIN1), *Haemoproteus pastoris* (lineage hLAMPUR01), but not of the lineage pGRW6 [2]. It is worth noting that Common startling is an invasive species spreading globally [23] in areas where *P. elongatum* has been often reported [2]. Common crossbills are also broadly distributed in the Holarctic zoogeographical region where transmission of *P. elongatum* also takes place. Both of these bird species likely have been exposed to this infection naturally and should be evolutionary adapted to this pathogen. A hypothesis was proposed that the lack of pGRW6 reports in crossbills and starlings might indicate their innate resistance to this infection. We aimed to test this hypothesis experimentally. *Plasmodium elongatum* (lineage pGRW6) was isolated from naturally infected reed warbler and experimentally passaged into juvenile starlings and crossbills, which were monitored in controlled laboratory conditions and examined using microscopic and histological methods.

## Methods

### Study site

Experimental work was carried out at the Biological Station of the Zoological Institute of the Russian Academy of Sciences on the Curonian Spit in the Baltic Sea (55°05′ N, 20°44′ E) in July and August of 2016. Mist nets and Rybachy-type funnel traps were used to catch juvenile wild birds (< 7 months old). All birds were screened for haemosporidian infections using microscopic examination and only the non-infected individuals were selected. Negative result of prior haemosporidian infections of experimental birds was later confirmed using polymerase chain reaction (PCR)-based screening methods in the laboratory.

### Experimental design

All birds were maintained under controlled conditions at a natural light–dark photoperiod. Control and experimental groups of each bird species were maintained in separate cages (size of 90 × 50 × 90 cm) located close to each other. The Common crossbills were maintained indoors in a vector-free room. The Common starlings were kept in an outside aviary, in cages, covered with a fine-mesh bolting silk preventing penetration of blood-sucking insects.

A strain of *P. elongatum* (lineage pGRW6, GenBank accession no. DQ368381), isolated from a naturally infected Eurasian reed warbler *Acrocephalus scirpaceus* was multiplied in two uninfected Eurasian reed warblers and used to infect the recipient birds of each species. 50 µl of infected donor blood was mixed with 12.5 µl sodium citrate and 62.5 µl of 0.9% saline solution per recipient bird [24]. The prepared mixture was sub-inoculated into the pectoral muscle of the experimental birds of both species. Each bird was inoculated approximately 125 µl of the inoculum. In all, 16 Common starlings and 16 Common crossbills were used for this study: 8 birds of each species were inoculated with same isolate of *P. elongatum* while the remaining 8 birds of each species were maintained as controls to prove the absence of natural transmission of haemosporidians during this experiment.

All birds were maintained for 44 days post exposure (DPE). Birds of experimental and control groups were weighed and blood from brachial vein was collected for microscopic examination and haematocrit level measures every 4 days. Brachial vein was punctured with a sterile needle and approximately 50 µl of blood was collected in heparinized microcapillaries. A small drop of blood was used to make three blood films, which were air-dried, fixed by dipping in absolute methanol for 3 min., stained with Giemsa and examined microscopically [12]. Approximately 20 µl of the blood in the microcapillary was fixed in SET buffer (0.05 M Tris, 0.15 M NaCl, 0.5 M EDTA, pH 8.0) for molecular analysis. These samples were maintained at − 4 °C in the field and later at − 20 °C in the laboratory. Remaining blood was used for centrifugation (10,000 rpm for 5 min.) and measuring of haematocrit value.

At the end of the experiment, all experimental birds were euthanized by decapitation, and the brain, heart, kidneys, liver, lungs, spleen, and a piece of the pectoral muscle were collected and fixed with 10% neutral formalin. Additionally, a smear of bone marrow was prepared; these smears were fixed with absolute methanol, stained with Giemsa keeping the same protocol as for blood films and examined using light microscope [12]. In the laboratory the collected tissues were embedded in paraffin blocks. Histological sections of 4 µm were prepared, stained with haematoxylin-eosin (H&E) and examined microscopically [12].

### Morphological analysis

An Olympus BX51 light microscope equipped with the Olympus DP12 digital camera and imaging software Olympus DP-SOFT were used to examine preparations. Each blood film was examined for 15–20 min. at medium magnification (×400), and then at least 100 fields were studied at high magnification (×1000). Intensity of parasitaemia was calculated as a percentage by actual counting of the number of parasites per 1000 erythrocytes or per 10,000 erythrocytes if infections were light [25]. Histological preparations were examined at low magnification (×200) for 10–15 min., followed by examination at medium magnification (×400) for 10–15 min. and then at high magnification (×1000) for another 20–30 min.

### Statistical analyses

Statistical analyses were carried out using the ‘R’ package [26]. Normality of data distribution was evaluated by applying the Shapiro–Wilk test. Differences between the means for data which were not distributed according to normal distribution were evaluated using the Wilcoxon test. Fisher’s exact test was used to evaluate if there was a statistically significant difference between haematocrit levels and body mass between the control and experimental groups in each bird species.

### Molecular analysis

Total deoxyribonucleic acid (DNA) was extracted from SET buffer fixed blood samples using an ammonium-acetate protocol [27]. Partial mitochondrial cytochrome *b* (*cytb*) sequences were amplified using a nested-PCR protocol [28, 29]. PCR mixes consisted of 12.5 µl of Dreamtaq Master Mix (Thermo Fisher Scientific, Lithuania), 8.5 µl of nuclease-free water, 1 µl of each primer and 2 µl of template DNA. Primer pair HaemFNI/HaemNR3 was used for the first PCR according the protocol described by [29]. For the second PCR, the primer pair HAEMF/HAEMR2 was used according to the protocol by [28]. For the second PCR instead of genomic DNA, 2 µl of the first PCR product was used. PCR success was evaluated by performing electrophoresis on a 2% agarose gel. 2 µl of the second PCR product was used for this evaluation. Nuclease-free water (negative control) and a *Plasmodium* sample, which was positive in previous testing (positive control) were used to determine possible false amplifications. No case of false amplification was found. Positive PCR products were sequenced from the 5′ end with the HAEMF primer [28] using dye terminator cycle sequencing (Big Dye). Sequencing was carried out using an ABI PRISM TM 3100 capillary sequencing robot (Applied Biosystems, USA). Sequences of parasites were edited and examined using the BioEdit program [30]. The ‘Basic Local Alignment Search Tool’ (megablast algorithm) was used to identify the amplified *cytb* sequences [31]. The ‘Basic Local Alignment Search Tool’ of the MalAvi database was used to double check the identified sequences [2].

### Phylogenetic analysis

Phylogenetic tree was constructed using partial sequences (479 bp) of the mitochondrial *cytb* gene. In all, 37 sequences of *Plasmodium* and 9 sequences of *Haemoproteus* were used. One sequence of *Leucocytozoon* sp. (lineage lSISKIN1) was used as outgroup. The Bayesian phylogenetic tree was constructed using MrBayes version 3.1 software [32]. The best fitting model of evolution (GTR) was selected by software MrModeltest 3.7 [33]. Analysis was run for a total of 10 million generations with a sample frequency of every 100th generation. Before the construction of the consensus tree, 25% of the initial trees were discarded as the ‘burn in’ period. The tree was visualized using the software FigTree v1.4.3 [34].

## Results

Both microscopic and molecular examinations showed that all birds used in this study were free of haemosporidian parasites prior to the experimental infections. All control birds remained uninfected during the entire study, indicating absence of transmission of haemosporidian parasites in captivity.

**Fig. 1:**
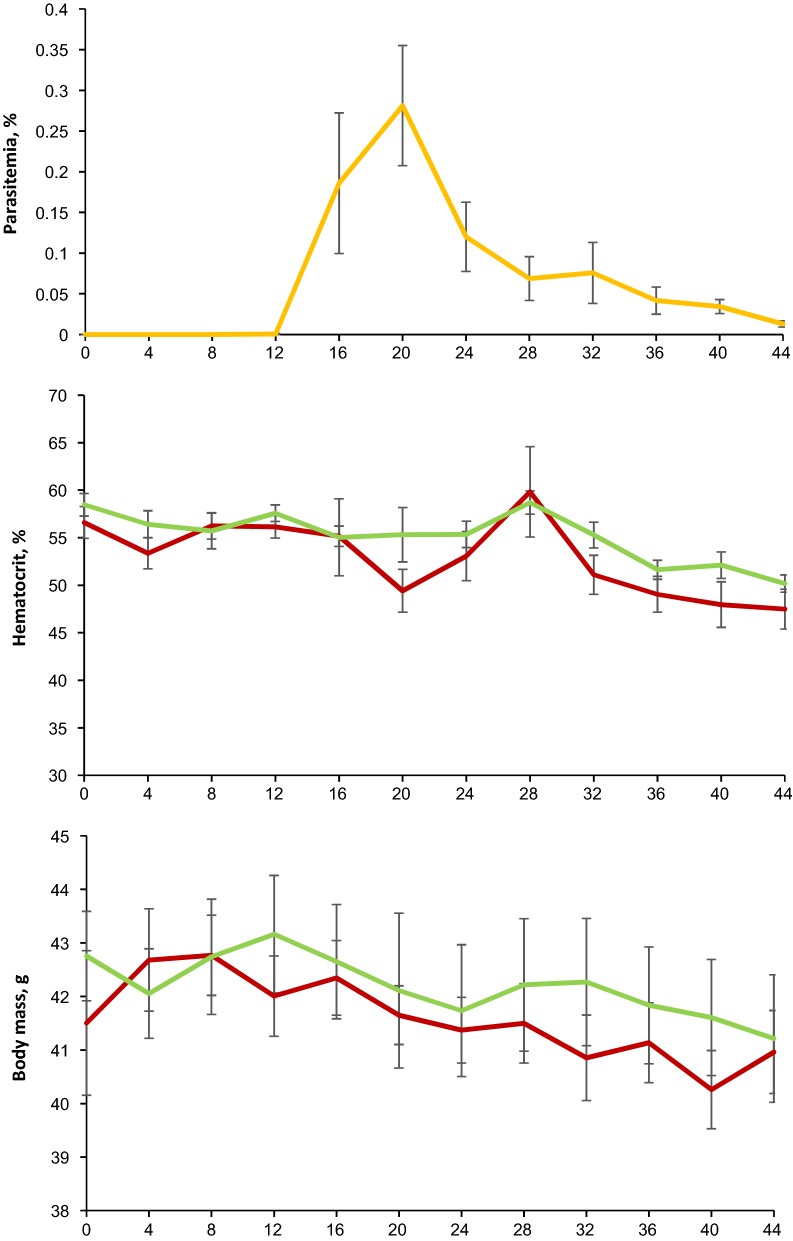
Dynamics of mean parasitaemia of *Plasmodium* (*Huffia*) *elongatum* (cytochrome *b* lineage pGRW6), mean haematocrit value and mean body mass in experimentally infected (red line) and control (green line) Common crossbills *Loxia curvirostra*. Abscissa shows days post exposure. Vertical lines indicate standard error

**Fig. 2:**
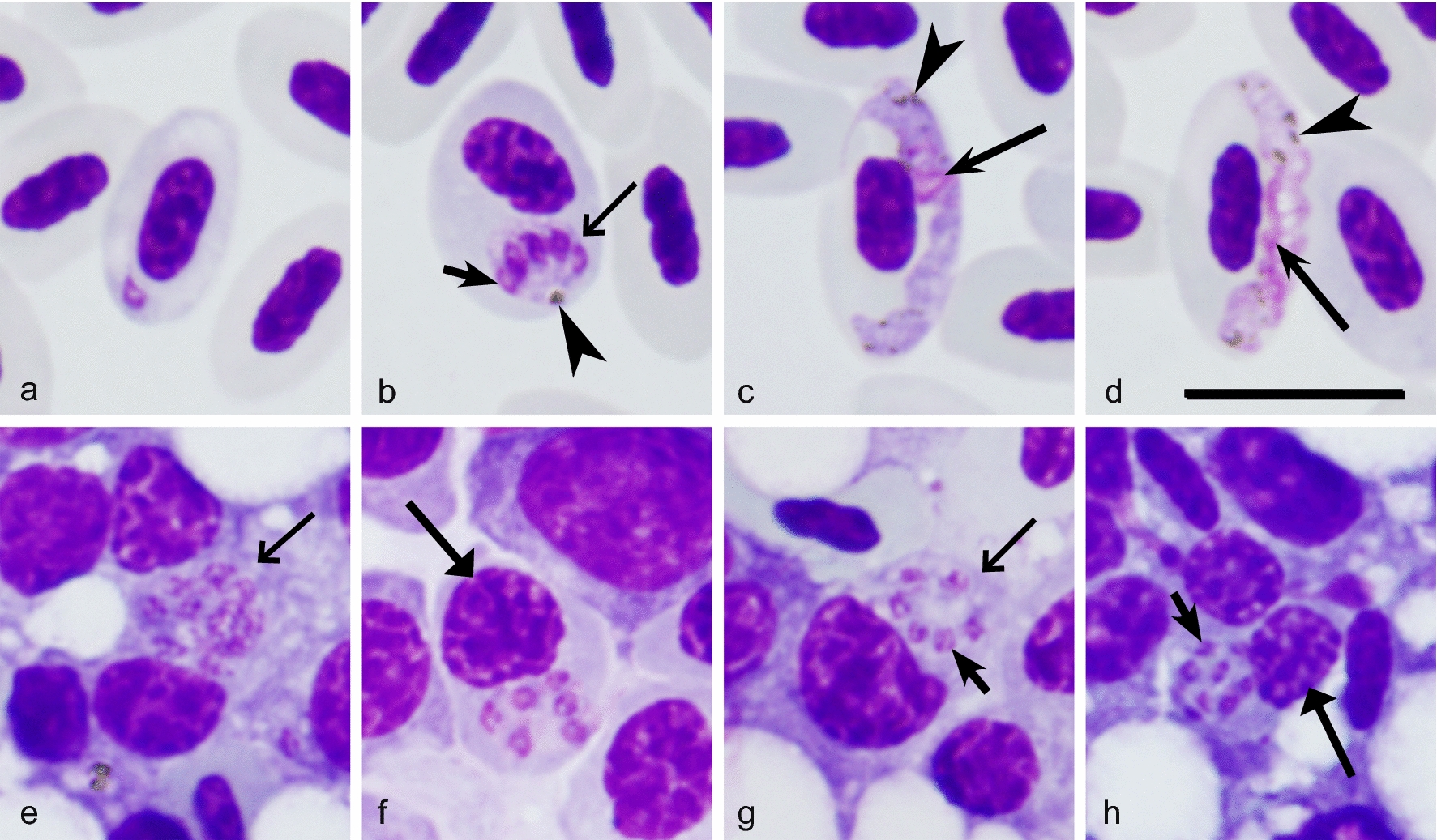
Blood stages (**a**–**d**) and bone marrow phanerozoites (**e**–**h**) of *Plasmodium* (*Huffia*) *elongatum* (cytochrome *b* lineage pGRW6) from experimentally infected Common crossbills *Loxia curvirostra*: **a**—trophozoite; **b**—erythrocytic meront; **c**—macrogametocyte; **d**—microgametocyte; **e**–**h**—secondary exoerythrocytic meronts (phanerozoites). Arrowheads (  )—pigment granules, long arrows with wide arrowheads (  )—meronts, short arrows (  )—merozoite, long arrows (  )—nuclei of the parasite, long arrow with triangle wide arrowhead (  )—nucleus of phanerozoite host cell. Note presence of meronts in immature erythrocytes (**b**), relatively small size of mature erythrocytic meronts (**b**), spindle-like shape of merozoites located in a parallel row in maturing erythrocytic meront (**b**), and attenuated elongate form of gametocytes (**c**, **d**). These are the main morphological characters of *P. elongatum*. Maturing phanerozoites are small (**f**–**h**), with bright blue cytoplasm, which texture has so much similarity to that of host cell that it is difficult to see line of separation between them. Pigment granules are absent from phanerozoites. Giemsa stained blood films. Scale bar = 10 µm

Microscopic examination showed that parasitaemia did not develop in any of the exposed Common starlings. That was confirmed by negative PCR tests in all experimental birds, indicating resistance of this avian host. All exposed Common crossbills were susceptible, with prepatent period ranging from 12 to 16 (on average 15) DPE (Fig. 1). Both microscopic (Fig. 2a–d) and PCR-based examinations showed presence of a single *P. elongatum* (lineage pGRW6) infection. Typical blood stages of *P. elongatum* were observed (Fig. 2a–d). Mainly, erythrocytic meronts occurred in immature erythrocytes; the parasites were small and contained readily visible pigment and elongate merozoites, which were arranged parallel to each other in a row (Fig. 2b). Gametocytes located in mature erythrocytes; the parasites were thin and elongate, with uneven margins and few small pigment granules present in the cytoplasm (Fig. 2c, d). Parasitaemia reached a small peak (0.3% average parasitaemia) on 20 DPE (Fig. 1). After this slight increase, the parasitaemia fluctuated in each individual bird, but did not reach the peak level again (Fig. 1). In spite of low parasitaemia, the decrease in average haematocrit value of the experimental group compared to the control group was significant (p < 0.001) (Fig. 1); that coincided with the small increase of parasitaemia (20 and 32 DPE) (Fig. 1). After the initial decrease (20 DPE), haematocrit value was restored and even surpassed the initial level, but after the next decrease (32 DPE) it remained low to the end of this study (Fig. 1).

No significant changes between the body mass of experimental and control groups of Common crossbills were observed during the experiment (p = 0.184) (Fig. 1). All exposed Common crossbills survived to the end of this study. 1, 2, 3, 5 and 55 phanerozoites were observed in the bone marrow only of 5 exposed bird individuals (Fig. 2 e–h). Phanerozoites were not seen in bone marrow preparations of 3 experimental birds, and they were not reported in other organs in all exposed birds.

**Fig. 3:**
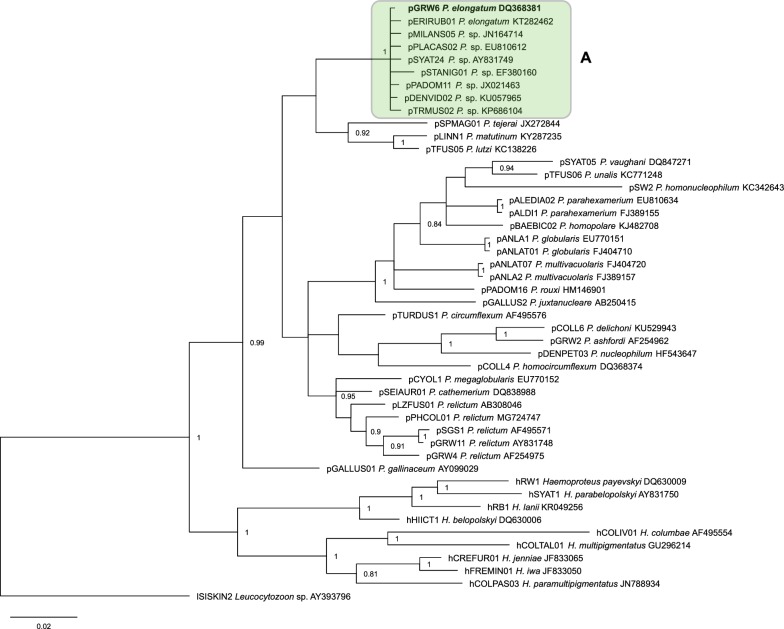
Bayesian phylogenetic tree constructed using 479 bp length mitochondrial cytochrome *b* gene fragments of 37 *Plasmodium* spp. and 9 *Haemoproteus* spp. lineages. One *Leucocytozoon* sp. lineage was used as an outgroup. Posterior probabilities of > 0.7 are indicated. MalAvi codes of lineages are given, followed by parasite species names and GenBank accession numbers for each sequence. Bold font indicates the *Plasmodium* (*Huffia*) *elongatum* pGRW6 lineage used in the present experimental study. Clade A indicates a group of closely related lineages, which all likely belong to *P. elongatum*

Phylogenetic analysis showed that *P. elongatum* (pGRW6) groups with the lineage pERIRUB01 belonging to the same parasite species as well as 6 non-identified closely related lineages, 6 of which differ from both identified lineages of *P. elongatum* only by 1 bp (Fig. 3).

## Discussion

This study supports the proposed hypothesis about innate resistance of Common starlings to the pGRW6 lineage of *P. elongatum*. This conclusion might explain why *P. elongatum* (pGRW6) has never been found in Common starlings worldwide [2]. This lineage of *P. elongatum* has been reported in birds belonging to 70 different species all over the world [2] thus, certainly it is a generalist malaria parasite. However, it is important to note that in the majority of these reports, *P. elongatum* (pGRW6) infection was detected using only molecular methods and it remains unclear if the parasite is able to complete its life cycle in all of the reported avian hosts to gametocyte stage, which is essential for natural transmission. This study indicates that some reports of *P. elongatum* (pGRW6) might be abortive infections, as is the case in the Common starlings, and PCR amplifications might be due to presence of circulating sporozoites, which are unable to initiate infections in resistant avian hosts [35]. Further studies are needed to specify this issue.

Interestingly, the Common starling is resistant or can tolerate several different malaria infections. For example, former experimental research with a cosmopolitan generalist parasite *P. relictum* (pSGS1) showed that this bird species was resistant as well [9]. *Plasmodium relictum* has been reported to infect a particularly big number of birds (> 300 species) [12, 36–38], however not a single report of the lineage pSGS1 or relative lineage pGRW11 have been from the Common starling so far. Additionally, a recent experimental study with *P. homocircumflexum* (lineage pCOLL4) showed that Common starlings were susceptible to this parasite and light parasitaemia developed, however, all individuals resisted the development of secondary exo-erythrocytic meronts (phanerozoites) resulting in low virulence and absence of any clinical signs of the infection [10].

*Plasmodium homocircumflexum* (lineage pCOLL4) is virulent and kills many bird species [10, 39, 40], but Common starlings readily tolerate this infection. Complete or partial resistance of Common starling to different species of avian malaria parasites may be one of the factors allowing this bird species to spread globally [41]. Mechanisms responsible for the Common starlings’ resistance or/and tolerance to infections of different species of avian *Plasmodium* remain unclear, and the mentioned above host-parasite model organisms could be used for research aiming at better understanding of the molecular mechanisms of the innate resistance during avian malaria.

Experiments with Common crossbills showed different results. Mainly, all exposed birds were susceptible and developed light parasitaemia. However, phanerozoites were reported in bone marrow preparations of only 63% of the infected individuals. In the majority of the phanerozoite-positive birds, only between 1 and 5 parasites were seen in bone marrow, indicating low secondary exo-erythrocytic development. Because phanerozoites often cause severe pathology in birds [10, 11, 15, 40] it seems that the ability to resist the development of the phanerozoites may explain the low virulence of *P. elongatum* pGRW6 in this bird species, which is readily susceptible to *P. relictum* (pSGS1), *P. homocircumflexum* (pCOLL4) and some other avian malaria parasites [9, 10, 15, 40].

Prepatent period of *P. elongatum* infection both in sporozoite and blood-induced infections varied between 9 and 12 days in experimentally exposed Domestic canaries and ducklings [11, 12]. This study in accord with these data, however parasitaemia was reported 16 DPE in 7 of 8 Common crossbill individuals. This was probably because blood was tested not daily, but every 4 DPE.

Because parasitaemia was readily recognizable in exposed Common crossbills, it is unclear why the *P. elongatum* (pGRW6) infection has not been reported in wild populations of Common crossbills before [2]. It might be due to the fact that the majority of published studies of haemosporidian parasites in crossbills were performed on the Curonian Spit during spring–summer migration or irruptions when mainly juvenile birds were sampled in May–June [8, 10, 40, 42]. This bird represents a unique ecological group of the Holarctic bird species that can breed in winter or in early spring [43] when mosquito vectors are inactive and transmission of malaria is absent, resulting in absence of malaria in Common crossbills during the sampling time [44]. This might explain the absence of *Plasmodium* and the paucity of other haemosporidian parasites in the 307 examined juvenile Common crossbills during the spring migration from the northern European breeding grounds to the southern latitudes on the Curonian Spit in June during a 3-year study [44]. Investigation of adult individuals in summertime is necessary to answer the question concerning the prevalence of *P. elongatum* in this bird species.

Former studies showed that *P. elongatum* parasitaemia is usually light [11, 12, 15, 45], and this is in accordance to this study. A small increase of parasitaemia was observed 20 DPE, however parasitaemia rapidly decreased and it was relatively stable during the remaining period of the observation, with gametocytes readily predominating in the circulation. Persistence by light parasitaemia might be an evolutionary adaptation, which should be beneficial for the parasite. Firstly, the impact of the parasites on the avian hosts is relatively low during light parasitaemia, and infected birds are more likely to survive [12, 46]. Experimental observations show that the exposed birds tend to be more active when they do not cope with symptoms of disease [47]. In other words, such avian hosts are less likely to be killed by predators, providing better opportunities for the parasite to be transmitted further. Secondly, high gametocytaemia might be virulent and have a negative effect on the vectors due to damage of their midgut by migrating ookinetes. However, data about the virulence of avian *Plasmodium* parasites on blood-sucking insects remain insufficient. Experimental observations showed that *Haemoproteus* species, a sister genus of haemosporidians, caused high mortality in mosquitoes and biting midges after infecting by blood meal with heavy gametocytaemia [48]. It is plausible that a similar effect might occur during the sporogonic development of *Plasmodium* parasites, therefore research should be conducted to test this hypothesis. Either way, low parasitaemia coupled with predominated gametocytaemia should contribute to parasite transmission from both, the hosts and the vectors, perspectives. That might explain why light parasitaemia of haemosporidian parasites predominate in the wild [47].

Haematocrit value dropped slightly during the small peak of parasitaemia, however it returned to normal levels several days after the peak when parasitaemia decreased (Fig. 1). The decrease in haematocrit level has been reported and is a common feature in other avian malaria infections during the parasitaemia peaks [9, 10, 49–51]. This is the first study which reports a decrease in haematocrit level during *P. elongatum* infection in relation to parasitaemia. It was believed that the decrease of haematocrit level during *P. elongatum* infections in domestic canaries and ducklings was mainly related to the damage caused by phanerozoites in the hematopoietic stem cells, which are responsible for erythropoiesis [2, 11, 15, 45]. It is important to note that, contrary to the infection of pERIRUB01 lineage of *P. elongatum* [15], few phanerozoites were observed in the bone marrow in this study. However, despite the low number or even absence of phanerozoites in the bone marrow of experimental birds, haematocrit value decreased slightly, but significantly compared to the control group (Fig. 1). These data indicate that the destruction of the infected erythrocytes also plays some role in the fluctuation of haematocrit value during *P. elongatum* infections, but it is not so obvious and is short-term in comparison to *P. relictum* [9, 52] or *P. homocircumflexum* [10, 40] infections. In other words, this study emphasizes that blood pathology also influences the haematocrit level fluctuations during *P. elongatum* infection.

One of the commonly accepted signs of degrading bird health is the decrease of the body mass during heavy haemosporidian infections, which might be accompanied with the decrease in host locomotion activity [12, 47]. However, recent studies show that there is no general pattern in regard of influence of different species of malaria parasites on the body mass of avian hosts: there are reports about not changed, decreased or even increased body mass in exposed birds compared to the control groups during different malarial infections [9, 10, 22]. Results of this study indicate that the changes in birds’ body mass is not a direct indicator of bird health, at least in some haemosporidian parasite infections.

*Plasmodium elongatum* is known to be highly virulent in non-adapted birds. There is a number of reports of this infection causing lethal diseases in penguins in zoos and rehabilitation centres all over the world [4–6, 16–21]. It is interesting to note that virulence of this parasite can vary in different hosts remarkably—from complete resistance (starlings, present study), to complete susceptibility and low virulence (crossbills, present study) and complete susceptibility and high virulence (species of penguins and the Brown kiwi, [4–6]). This raises a question about mechanisms, which might be responsible for these differences in the virulence: are they related to the host and their ability to resist infections or the parasite and its ability to infect the host, or a combination of both? Available experimental data show that Common starlings are able to fully resist two (*P. relictum* and *P. elongatum*) and partially resist one species (*P. homocircumflexum*) of avian malaria parasite species [9, 10, present study]. It seems that this host species is able to cope with malaria infections. However, it remains unclear what mechanisms are responsible for this feature. Common crossbills, Eurasian siskins and Domestic canaries have been repeatedly shown to be good model organisms for avian malaria research [15, 53–55] as they are susceptible to many species of avian malaria and are seemingly genetically different from Common starlings in regard to the ability to resist avian malaria. Similar situation is in the case of the Magellanic penguin (*Spheniscus magellanicus*) and many other penguin species; based on the available data these birds can host different lineages of haemosporidian parasites [2], suggesting they might lack the resistance to these parasites. Based on the available molecular data, it is clear that *P. elongatum* is able to infect various avian hosts [2, 12]. The cosmopolitan *P. elongatum* and its various vertebrate hosts, in which the same lineage develops differently, is a good model system to access molecular mechanisms of resistance during avian malaria.

A group of lineages, which are closely related to *P. elongatum* (pGRW6) appeared in one well-supported clade in the phylogenetic tree (Fig. 3, clade A). Because genetic difference between these lineages is negligible, it is probable that all these parasites are intraspecies variants of *P. elongatum*. Morphological evidence is needed to prove this hypothesis because some readily distinguishable haemosporidian species are very similar in *cytb* gene partial sequence and can differ just in few nucleotides [29, 56]. Only two lineages (pGRW6 and pERIRUB01) have been linked to this species so far. Because the lineages pERIRUB01 and pGRW6 are closely related and genetically similar in *cytb* but are different in regard of their virulence and ability to develop phanerozoites [15, this study], it is predictable that the lineages of the clade A also might be different in their biological features and the ability to cause disease in birds. Intraspecies variation in *cytb* gene is a common feature in avian haemosporidian parasites but remains insufficiently investigated in regard of the biology of parasites of these lineages. In other words, the biological meaning of negligible difference between closely related lineages of the same parasite morphospecies remain insufficiently understood. Due to the global distribution and the easy morphological species identification using blood stages, the parasites of different lineages of *P. elongatum* are convenient model organisms for research aiming at better understanding the virulence and other biological features of the parasites representing the closely related intraspecies variants of the same *Plasmodium* species. For example, the early studies of *P. elongatum* showed that the American and European isolates of same species are markedly different in the ability to cause severe disease in domestic canaries, but the genetic characterization was insufficiently developed at that time [11]. Recent molecular techniques provide opportunities for easier distinguishing and genetic characterization of different *P. elongatum* strains and opens opportunities for targeting experimental research on the biology of malaria parasites on their lineage levels.

## Conclusion

The key result of this study is that the susceptibility of different bird species to the same *cytb* lineage of *P. elongatum* varies markedly. It might manifest itself from complete resistance to complete susceptibility with the ability to tolerate the infection as well as complete susceptibility with severe disease and high mortality. This should be taken into consideration in bird management and veterinary medicine. The global distribution of *P. elongatum* (pGRW6) might be due to the low virulence in some species of wild birds, which readily survive this infection, but serve as reservoirs hosts for non-adapted birds, in whom this parasite is often lethal. The complete resistance or ability to tolerate infections of common malaria agents in some avian hosts should be considered as the factors contributing to spread of invasive birds globally. This is likely the case with Common starlings and *P. elongatum* (pGRW6), and worth attention in biogeography and epidemiology, particularly of invasive species of hosts and parasites.

## Data Availability

The datasets used and/or analyzed during the current study are available from the corresponding author on reasonable request.

## Abbreviations

*cytb*: mitochondrial cytochrome *b*
DNA: deoxynucleic acid
DPE: days post exposure
H&E: haematoxylin–eosin
PCR: polymerase chain reaction
